# Supplementary material for: Association of Chocolate Consumption with Hearing Loss and Tinnitus in Middle-Aged People Based on the Korean National Health and Nutrition Examination Survey 2012–2013
Source: Nutrients. 2019 Mar 30;11(4):746. doi: 10.3390/nu11040746 (PMC6520725; doi:10.3390/nu11040746)
Supplement: Supplementary file 1 [file nutrients-11-00746-s001.pdf]

Table S1. Assessment of chocolate consumption Food-Frequency Questionnaire (FFQ)

Question 1. What was the average frequency of intake of chocolate over the last year?
1. Never or seldom
2. Once per month
3. Two to three times per month
4. Once per week
5. Two to four times per week
6. Five to six times per week
7. Once per day
8. Twice per day
9. Three times per day
10. No response

Question 2. What is your average intake of chocolate?
1. Rarely (less than once per month)
2. One quarter of a chocolate tablet
3. One half of a chocolate tablet
4. One chocolate tablet
5. No response

Supplement table 2. Subgroup analysis regarding effect of chocolate on hearing loss and tinnitus according to sex

	**chocolate interaction**
**Any hearing loss (unilateral or bilateral)**	0.8822
**Hearing loss (bilateral)**	0.0567
**Tinnitus**	0.2667
**Tinnitus-related annoyance**	0.4143

	**Estimate**	**95% CI**		**P-value**	**Estimate**	**95% CI**		**P-value**
		**Lower**	**Upper**			**Lower**	**Upper**	
**Men**								
**Any hearing loss (unilateral or bilateral)**	0.652	0.521	0.817	0.0002	0.852	0.662	1.098	0.2158
**Hearing loss (bilateral)**	0.545	0.418	0.712	<.0001	0.719	0.536	0.965	0.0282
**Tinnitus**	0.844	0.64	1.113	0.23	0.924	0.691	1.237	0.5973
**Tinnitus-related annoyance**	0.57	0.355	0.914	0.0196	0.709	0.432	1.166	0.1753
**Women**								
**Any hearing loss (unilateral or bilateral)**	0.642	0.521	0.791	<.0001	0.823	0.656	1.033	0.0938
**Hearing loss (bilateral)**	0.665	0.502	0.88	0.0044	0.886	0.654	1.2	0.434
**Tinnitus**	0.876	0.712	1.078	0.2123	0.902	0.728	1.117	0.3438
**Tinnitus-related annoyance**	0.925	0.658	1.3	0.6538	1.022	0.719	1.452	0.9042
